# Supplementary material for: Associations between Nutritional and Immune Status and Clinicopathologic Factors in Patients with Pancreatic Cancer: A Comprehensive Analysis
Source: Cancers (Basel). 2021 Oct 9;13(20):5041. doi: 10.3390/cancers13205041 (PMC8533745; doi:10.3390/cancers13205041)
Supplement: Supplementary file 1 [file cancers-13-05041-s001.zip › cancers-1367776-supplementary.pdf]

Table S1. The patient general clinical characteristics.

Feature	Value
Demographic characteristics	
Age (years)	65.44 ± 8.37 (41-86)
≤ 65 years	39 (49.75 %)
> 65 years	41 (51.25 %)
Male/Female	40 (50.00 %) / 40 (50.00 %)
Weight loss %	
Stable	49 (61.25 %)
Weight loss ≤ 10%	12 (15.00 %)
Weight loss > 10%	19 (23.75 %)
Weight [kg]	70.92 ± 12.92 (40.00-99.00)
Weight loss [kg]	7.77 ± 8.33 (0.00-30.00)
BMI (kg/m²)	25.04 ± 3.51 (16.64-34.25)
BMI groups according to WHO classification	
< 18.5	3 (3.75 %)
18.5-24.9	33 (41.25 %)
25-29.9	39 (48.75 %)
≥ 30	5 (6.25 %)
BMI general division	
< 18.5	3 (3.75 %)
> 18.5	77 (96.25 %)
NRS 2002 (points)	2.14 ± 1.11 (1-5)
NRS 2002 classification	
0	0 (0.00 %)
1	25 (31.25 %)
2	34 (42.50 %)
3	10 (12.50 %)
4	7 (8.75 %)
5	4 (5.00 %)
NRS 2002 groups	
< 3 points	59 (73.75 %)
≥ 3 points	21 (26.25 %)
Arterial hypertension	
No	40 (50.00 %)
Yes	40 (50.00 %)
Ischemic heart disease	
No	75 (93.75 %)
Yes	5 (6.25 %)
Arterial fibrillation	
No	76 (95.00 %)
Yes	4 (5.00 %)
Other heart arrhythmia	
No	73 (91.25 %)
Yes	7 (8.75 %)
Diabetes mellitus (type 1)	
No	77 (96.25 %)
Yes	3 (3.75 %)
Diabetes mellitus (type 2)	

No	55 (68.75 %)
Yes	25 (31.25 %)
Pulmonary obturative disease /asthma	
No	72 (90.00 %)
Yes	8 (10.00 %)
Smoking	
No	48 (60.00 %)
Yes	32 (40.00 %)
Smoking cessation	
No	14 (17.50 %)
Yes	18 (22.50 %)
Not applicable	48 (60.00 %)
Duration of smoking (years)	5 ± 12.18 (5-53)
Number of cigarettes per day	18.85 ± 3.45 (10-20)
Alcohol consumption	
No	72 (90.00 %)
Yes	8 (10.00 %)
Preoperative biliary stenting	
No	33 (41.25 %)
Yes	47 (58.75 %)
Duration between biliary stenting and operation [months]	3.44 ± 3.57 (0.50-18.00)
Previous other operations	
No	53 (66.25 %)
Yes	27 (33.75 %)
Clinical manifestation	
Jaundice	42 (52.50 %)
Abdominal pain	37 (46.25 %)
Diarrhea	13 (16.25 %)
Nausea	9 (11.25 %)
Vomitus	6 (7.50 %)
Acute pancreatitis	5 (6.25 %)
Loss of appetite	4 (5.00 %)
Meteorism	4 (5.0 %)
Weakness	3 (3.75 %)
Flipping	2 (2.50 %)
New diabetes	2 (2.50 %)
Constipation	2 (2.50 %)
Fever	1 (1.25 %)
Cholangitis	1 (1.25 %)
Duration of clinical symptoms [months]	4.95 ± 3.90 (1-18)
Duration of weight loss [months]	4.02 ± 3.06 (0.5-12.00)
BMI, body mass index; NRS , Nutritional Risk Score	

Table S2. The patients clinical characteristics regarding hospitalization and surgery.

Feature	Value
Total duration of hospitalization [days]	14.77 ± 8.90 (6-72)
Postoperative duration of hospitalization [days]	12.97 ± 8.89 (5-71)
Hospitalization in ICU	
No	67 (83.75 %)

Yes	13 (16.25 %)
Duration of hospitalization in ICU	4.50 ± 6.29 (1-22)
General tumor location	
Proximal (head)	58 (72.50 %)
Distal (body, tail)	22 (27.50 %)
Detailed tumor location	
Head	58 (72.50 %)
Body	9 (11.25 %)
Tail	8 (10.00 %)
Body and tail	5 (6.25 %)
Types of surgical resection	
Proximal (pancreaticoduodenectomy)	55 (68.75 %)
Whipple	29 (36.25 %)
Traverso	26 (32.50 %)
Distal with splenectomy (RAMPS)	22 (27.50 %)
Total pancreatectomy	3 (3.75 %)
Duration of operation [min]	449.79 ± 127.72 (185-704)
ASA (points)	2.51 ± 0.53 (1-3)
ASA classification	
1	1 (1.25 %)
2	37 (46.25 %)
3	42 (52.50 %)
4	0 (0.00 %)
5	0 (0.00 %)
Blood loss [ml]	528.22 ± 340.28 (100-1600)
Blood transfusion	
No	66 (82.50 %)
Yes	14 (17.50 %)
Postoperative morbidity	18 (22.50 %)
Postoperative mortality	1 (1.25 %)
Reoperations	9 (11.25 %)
Readmissions	5 (6.25 %)
Types of postoperative complications	
POPF	4 (5.00 %)
B	2 (2.50 %)
C	2 (2.50 %)
Wound infection	4 (5.00 %)
Wound dehiscence (eventration)	2 (2.50 %)
Intraabdominal hematoma	3 (3.75 %)
Intraabdominal hemorrhage	1 (1.25 %)
Intraabdominal collection / abscess	4 (5.00 %)
Perforation of transverse colon / peritonitis	1 (1.25 %)
Sigmoid colon necrosis	1 (1.25 %)
Acute pancreatitis	2 (2.50 %)
DGE	2 (2.50 %)
Hydrothorax	6 (7.50 %)
Pneumonia	3 (3.75 %)
Pneumothorax	1 (1.25 %)
Covid-19 infection	1 (1.25 %)
Types of postoperative complications according to Clavien Dindo classification	

0	
1	62 (77.50 %)
2	7 (8.75 %)
3	2 (2.50 %)
4	8 (10.00 %)
5	0 (0.00 %)
	1 (1.25 %)
Surgical margin status	
R0	44 (55.00 %)
R1	17 (21.25 %)
R1 direct	16 (20.00 %)
R2	3 (2.75 %)
Neoadjuvant chemotherapy	
No	68 (85.00 %)
Yes	12 (15.00 %)
Chemotherapy regimen	FOLFIRINOX 12 (100 %)
Number of cycles	6.92 ± 2.94 (5-15)
Neoadjuvant radiotherapy	
No	79 (89.75 %)
Yes	1 1 (1.25 %)
Perioperative parenteral nutrition	
No	66 (82.50 %)
Yes	14 (17.50 %)
Duration of parenteral nutrition [days]	10.07 ± 5.27 (1-22)

ICU, Intensive Care Unit; ASA, American Society of Anesthesiologists; R, resection margin type; POPF, postoperative pancreatic fistula; DGE, delayed gastric emptying.

Table S3. Laboratory results.

**Feature**	**Value**
Total protein [g/dl]	6.10 ± 0.97 (3.90-7.40)
Albumin [g/dl]	3.50 ± 0.73 (2.0-4.7)
White blood cell count [/mm³]	7.40 ± 2.17 (2.81-13.70)
Neutrophil count [/mm³]	4.46 ± 161 (0.62-8.18)
Monocyte count [/mm³]	0.62 ± 0.25 (0.19-1.52)
Total lymphocyte count [/mm³]	1.93 ± 0.77 (0.61-4.24)
Platelet count[/mm³]	255.16 ± 102.83 (94.00-579.00)
Hemoglobin[g/dl]	13.08 ± 1.53 (9.75-16.10)
CRP [mg/l]	7.24 ± 10.60 (0.30-53.30)
ALT [U/l]	36.29 ± 19.99 (9.50-88.00)
AST [U/l]	40.14 ± 34.71 (17-200)
GGT [U/l]	63.67 ± 48.69 (2.00-172.00)
ALP [U/l]	119.93 ± 46.66 (49.00-220.00)
Bilirubin [g/dl]	1.13 ± 0.78 (0.36-3.91)
Creatinine [g/dl]	0.84 ± 0.17 (0.36-1.46)
Amylase [U/l]	53.31 ± 31.61 (13.00-147.00)
Lipase [U/l]	80.68 ± 92.03 (1.00-332.00)
Cholesterol [mg/dl]	169.68 ± 78.63 (34.00-371.00)
Triglycerides [mg/dl]	190.31± (66-514.00)
Prothrombin time [s]	12.22 ± 1.26 (9.80-17.60)
Prothrombin activity [%]	90.14 ± 13.82 (29.00-124.00)

APTT [s]	30.72 ± 5.03 (19.10-47.20)
INR	1.08 ± 0.11 (0.87-1.54)
CEA [ng/ml]	7.32 ± 10.33 (1.04-44.73)
CA 19.9 [U/ml]	392.99 ± 566.00 (0.75-1926.26)
Fluid amylase [U/l]	1862 ± 4323.03 (3-23871)
PNI	45.03 ± 8.42 (25.05-63.20)
NLR	2.92 ± 1.97 (0.25-11.12)
MLR	0.44 ± 0.45 (0.06-3.11)
PLR	156.81 ± 95.84 (41.67-500.00)

ALT; alanine aminotransferase; AST, aspartate aminotransferase; GGT; gamma-glutamyl transpeptidase; ALP, alkaline phosphatase; CRP, C-reactive protein; APTT; activated partial thromboplastin time; INR; international normalized ratio; CEA, carcinoembryonic antigen; CA 19.9, carbohydrate antigen; PNI, prognostic nutritional index; NLR, Neutrophil/lymphocyte ratio; MLR, Monocyte/lymphocyte ratio; PLR, Platelet/lymphocyte ratio.

Table S4. The tumor pathological characteristics.

Feature	Value
Tumor diameter [cm]	3.05 ± 1.01 (0.8-7.0)
Tumor depth (T) general division	
T1-2	71 (88.75 %)
T3	9 (11.25 %)
Tumor depth (T) detailed division	
T1	13 (16.25 %)
T2	58 (72.50 %)
T3	9 (11.25 %)
Lymph node invasion (N) general division	
N0	9 (11.25 %)
N+	71 (88.75 %)
Lymph node invasion (N) detailed division	
N0	9 (11.25 %)
N1	22 (27.50 %)
N2	49 (61.25 %)
Distant metastasis	
M0	71 (88.75 %)
M1	9 (11.25 %)
Histological type	
Adenocarcinoma	75 (93.75 %)
Adenosquamous carcinoma	5 (6.25 %)
Histological grading general division	
G1-2	58 (72.50 %)
G3	22 (27.50 %)
Histological grading detailed division	
G1	8 (10.00 %)
G2	50 (62.50 %)
G3	22 (27.50 %)
Microvascular invasion	
No	24 (30.00 %)
Yes	56 (70.00 %)
Perineural infiltration	
No	7 (8.75 %)
Yes	73 (91.25 %)

Immunohistochemistry	
AbPaS (+)	40 (50.00 %)
p63 (+)	12 (15.00 %)
Tumor regression score after neoadjuvant treatment	
1	0 (0.00 %)
2	7 (8.75 %)
3	5 (6.25 %)
Not applicable	68 (85.00 %)
Coexistent neoplasm	
IPMN (Intraductal papillary mucinous neoplasm)	7 (8.75 %)
MCN (Mucinous cystic neoplasm)	1 (1.25 %)
NET (Neuroendocrine tumor)	1 (1.25 %)
Absent	71 (88.75 %)

Table S5. Comparison of selected clinicopathological and nutritional parameters depending on the tumor location.

**Feature**	**Proximal location (pancreatic head) (*n* = 58)**	**Distal location (pancreatic body / tail) (*n* = 22)**	***p* value**
Age [years]	64.29 ± 7.95	68.45 ± 8.90	0.0465
Gender	30 (51.72 %) Male<br>28 (48.28 %) Female	10 (45.45 %) Male<br>12 (54.55 %) Female	0.6165
Tumor depth (T)	10 (17.24 %) T1<br>45 (77.59 %) T2<br>3 (5.17 %) T3	3 (13.64 %) T1<br>13 (59.09 %) T2<br>6 (27.27 %) T3	0.0202
Lymph node invasion (N)	6 (10.34 %) N0<br>15 (25.86 %) N1<br>37 (63.79 %) N2	3 (13.64 %) N0<br>7 (31.83 %) N1<br>12 (54.55 %) N2	0.7475
Distal metastasis (M)	53 (92.98 %) M0<br>4 (7.02 %) M1	18 (81.82 %) M0<br>4 (18.18 %) M1	0.1404
Histological type			
1. Adenocarcinoma	56 (96.55 %)	19 (86.36 %)	0.0928
1. Adenosquamous carcinoma	2 (3.45 %)	3 (13.64 %)	
Histological grading	4 (6.90 %) G1<br>38 (65.52 %) G2<br>16 (27.59 %) G3	4 (18.18 %) G1<br>12 (54.55 %) G2<br>6 (27.27 %) G3	0.3105
Weight [kg]	70.24 ± 12.51	72.70 ± 14.07	0.4498
Weight loss [kg]	9.37 ± 8.55	2.50 ± 4.88	0.0104
Weight loss			
1. Stable	31 (54.35 %)	18 (81.82 %)	0.0326
2. Weight loss ≤ 10 %	9 (15.52 %)	3 (13.64 %)	
3. Weight loss > 10 %	18 (31.03 %)	1 (5.55 %)	
BMI groups according to WHO classification<br>< 18.5<br>18.5-24.9<br>25-29.9<br>≥ 30	3 (5.17 %)<br>24 (41.38 %)<br>28 (48.28 %)<br>3 (5.17 %)	0 (0.00 %)<br>9 (40.91 %)<br>11 (50.00 %)<br>2 (9.09 %)	0.6730
Duration of clinical manifestation [months]	4.54 ± 3.79	7.37 ± 3.89	0.0482
NRS 2002 classification			
1.	18 (31.03 %	7 (31.82 %)	0.4852

2.	22 (37.93 %)	12 (54.55 %)	
3.	8 (13.79 %)	2 (9.09 %)	
4.	6 (10.34 %)	1 (4.55 %)	
5.	4 (6.90 %)	0 (0.00 %)	
Common clinical symptoms			
Jaundice	42 (72.41 %)	0 (0.00 %)	< 0.0001
Diarrhea	12 (20.69 %)	1 (4.55 %)	0.0805
Abdominal pain	28 (48.28 %)	9 (40.91 %)	0.5551
Neoadjuvant chemotherapy	9 (15.52 %)	3 (13.64 %)	0.8334
Count of chemotherapy cycles	6.44 ± 1.59	8.33 ± 5.77	0.7115
Preoperative biliary stenting	11 (18.97 %) No<br>47 (87.03 %) Yes	22 (100. 00 %) No<br>0 (0.00 %) Yes	<0.0001
Duration of hospitalization [days]	16.07 ± 9.55	11.36 ± 5.84	0.0002
Postoperative morbidity rate	14 (25.93 %)	4 (19.05 %)	0.5311
Postoperative mortality rate	1 (11.11 %)	0 (0.00 %)	0.3359
Reoperation rate	7 (12.07 %)	2 (9.09 %)	0.7067
Readmission rate	3 (5.26 %)	2 (9.09 %)	0.5311
Duration of operation	502.21 ± 94.67	311.59 ± 97.19	<0.0001
Blood loss [ml]	524.42 ± 313.84	536.84 ± 403.06	0.8786
ASA classification			
1.	1 (1.72 %)	0 (0.00 %)	0.6645
2.	28 (48.28 %)	9 (40.91 %)	
3.	29 (50.00 %)	13 (59.09 %)	
Smoking	37 (63.79 %) No<br>21 (36.21 %) Yes	11 (50.00 %) No<br>11 (50 %) Yes	0.2608
Postoperative parenteral nutrition	45 (77.59 %) No<br>13 (22.41 %) Yes	21 (95.45 %) No<br>1 (4.55 %) Yes	0.0373
Duration of parenteral nutrition [days]	7.63 ± 6.52	2.00 ± 3.46	0.1653
Total protein [g/dl]	5.71 ± 1.15	6.15 ± 0.98	0.4833
Albumin [g/dl]	3.42 ± 0.73	3.73 ± 0.70	0.0677
White blood cell count [/mm^3]	7.78 ± 2.12	6.41 ± 2.00	0.0109
Neutrophil count [/mm^3]	4.67 ± 1.47	3.79 ± 1.88	0.0865
Monocyte count [/mm^3]	0.63 ± 0.67	0.59 ± 019	0.5366
Total lymphocyte count [/mm^3]	1.99 ± 0.85	1.78 ± 0.49	0.5055
Platelet count[/mm^3]	270.00 ± 105.73	216.71 ± 85.56	0.0202
Hemoglobin [g/dl]	12.96 ± 1.50	13.42 ± 1.59	0.2326
CRP [mg/l]	7.89 ± 10.38	5.25 ± 11.41	0.0397
ALT [U/l]	41.56 ± 19.92	23.35 ± 13.58	0.0013
AST [U/l]	44.92 ± 39.21	26.69 ± 7.57	0.0971
GGT [U/l]	78.48 ± 47.03	30.37 ± 35.55	0.0008
ALP [U/l]	136.41 ± 42.69	74.62 ± 18.73	0.0008
Bilirubin [g/dl]	1.30 ± 0.86	0.70 ± 0.22	0.0162
Ca 19.9	286.15 ± 456.77	927.22 ± 865.36	0.0756
CEA [ng/ml]	4.80 ± 5.26	20.76 ± 20.79	0.0292
PNI	44.38 ± 8.63	46.90 ± 7.77	0.3221
NLR	2.74 ± 1.61	2.21 ± 1.05	0.4989
MLR	0.36 ± 0.16	0.37 ± 0.25	0.5820
PLR	158.78 ± 91.38	151.16 ± 110.91	0.5699

NRS 2002, Nutritional Risk Score; BMI, body mass index; ASA, American Society of Anesthesiologists; ALT; alanine aminotransferase; AST, aspartate aminotransferase; GGT; gamma-glutamyl transpeptidase; ALP, alkaline phosphatase; CRP, C-reactive protein; CEA, carcinoembryonic antigen; CA 19.9, carbohydrate antigen; PNI, prognostic nutritional index; NLR, Neutrophil/lymphocyte ratio; MLR, Monocyte/lymphocyte ratio; PLR, Platelet/lymphocyte ratio. Significant results ($p < 0.05$) are highlighted in red print.

Table S6. Comparison of selected clinicopathological and nutritional parameters depending on age.

Feature	Age ≤ 65 years (*n* = 39)	Age > 65 years (*n* = 41)	*p* value
NRS 2002	1.87 ± 1.00	2.39 ± 1.16	0.0326
ASA	2.38 ± 0.54	2.63 ± 0.49	0.0335
Weight [kg]	76.24 ± 11.47	65.86 ± 12.27	0.0002
Weight loss [kg]	8.70 ± 9.05	6.96 ± 9.77	0.5922
Total protein [g/dl]	6.36 ± 0.80	5.86 ± 1.05	0.0447
Albumin [g/dl]	3.68 ± 0.62	3.33 ± 0.79	0.0468
Total lymphocyte count [/mm^3]	1.98 ± 0.82	1.90 ± 0.75	0.8938
Neutrophil count [/mm^3]	4.75 ± 1.55	4.23 ± 1.64	0.2856
Monocyte count [/mm^3]	0.59 ± 0.21	0.64 ± 0.27	0.9878
White blood cell count [/mm^3]	7.46 ± 2.14	7.35 ± 2.20	0.8228
Platelet count [/mm^3]	251.53 ± 106.95	258.53 ± 100.09	0.5462
Hemoglobin [g/dl]	13.41 ± 1.54	12.78 ± 1.47	0.0265
CRP [mg/l]	7.38 ± 10.52	7.12 ± 10.85	1.0000
PNI	47.77 ± ±7.62	42.96 ± 8.52	0.0300
NLR	2.62 ± 1.27	2.60 ± 1.68	0.6785
MLR	0.33 ± 0.13	0.38 ± 0.22	0.8016
PLR	149.27 ± 83.33	162.52 ± 102.13	0.6153
Postoperative parenteral nutrition	36 (92.31 %) No<br>3 (7.69 %) Yes	30 (73.17 %) No<br>11 (26.83 %) Yes	0.0115
Duration of parenteral nutrition [days]	3.28 ± 5.00	10.45 ± 5.82	0.0243
Duration of hospitalization	12.87 ± 4.37	16.58 ± 11.47	0.2073
Postoperative morbidity	7 (18.92 %)	11 (28.95 %)	0.3076
Postoperative mortality	0 (0.00 %)	1 (2.44 %)	0.5125
Reoperation rate	2 (5.13 %)	7 (17.07 %)	0.0894
Readmission rate	1 (2.63 %)	4 (9.76 %)	0.2040
Arterial hypertension	20 (51.28 %) No<br>19 (48.72 %) Yes	20 (48.78 %) No<br>21 (51.22 %) Yes	0.8230
Ischemic heart disease	37 (94.87 %) No<br>2 (5.13 %) Yes	38 (92.68 %) No<br>3 (7.32 %) Yes	0.5240
Diabetes mellitus (type 2)	27 (69.23 %) No<br>12 (30.77 %) Yes	28 (68.29 %) No<br>13 (31.71 %) Yes	0.9279

NRS 2002, Nutritional Risk Score; BMI, body mass index; ASA, American Society of Anesthesiologists; ICU, Intensive Care Unit; ALT; alanine aminotransferase; AST, aspartate aminotransferase; GGT; gamma-glutamyl transpeptidase; ALP, alkaline phosphatase; CRP, C-reactive protein; APTT; activated partial thromboplastin time; INR; international normalized ratio; CEA, carcinoembryonic antigen; CA 19.9, carbohydrate antigen; PNI, prognostic nutritional index; NLR, Neutrophil/lymphocyte ratio; MLR, Monocyte/lymphocyte ratio; PLR, Platelet/lymphocyte ratio. Significant results ($p < 0.05$) are highlighted in red print.

Table S7. Comparison of selected clinicopathological and nutritional parameters depending on the NRS 2002 classification.

Feature	NRS 2002 < 3 (*n* = 59)	NRS 2002 ≥ 3 (*n* = 21)	*p* value

Age [years]	64.61 ± 8.71	67.76 ± 7.01	0.1396
Age groups<br>≤ 65 years<br>> 65 years	33 (55.93 %)<br>26 (44.07 %)	6 (28.57 %)<br>15 (71.43%)	0.0312
Gender	31 (52.54 %) Male<br>28 (47.46 %) Female	9 (42.85 %) Male<br>12 (57.14 %) Female	0.3060
General tumor location	40 (67.80 %) Proximal<br>19 (32.20 %) Distal	18 (87.71 %) Proximal<br>3 (14.29 %) Distal	0.0946
Detailed tumor location	40 (67.80 %) head<br>7 (11.86 %) body<br>7 (11.86 %) tail<br>5 (8.47 %) body and tail	18 (85.71 %) head<br>2 (9.52 %) body<br>1 (4.76 %) tail<br>0 (0.00 %) body and tail	0.1956
Tumor depth (T)	7 (18.76 %) T1<br>46 (77.97 %) T2<br>6 (10.17 %) T3	6 (28.57 %) T1<br>12 (57.14 %) T2<br>3 (14.29 %) T3	0.1481
	26 (44.07 %) T1-2<br>33 (55.93 %) T3	7 (33.33 %) T1-2<br>14 (66.67 %) T3	0.2762
Lymph node invasion (N)	6 (10.17 %) N0<br>17 (28.81 %) N1<br>36 (61.02 %) N2	3 (14.29 %) N0<br>5 (23.81 %) N1<br>13 (61.90 %) N2	0.8285
	6 (10.17 %) N0<br>53 (89.83 %) N +	3 (14.29 %) N0<br>18 (85.71 %) N +	0.4361
Distal metastasis (M)	54 (93.10 %)<br>4 (6.90 %)	17 (80.95 %)<br>4 (19.05 %)	0.1138
Histological type<br>2. Adenocarcinoma<br>3. Adenosquamous carcinoma	55 (93.22 %)<br>4 (6.78 %)	20 (95.24 %)<br>1 (4.76 %)	0.6058
Histological grading	8 (13.56 %) G1<br>35 (59.32 %) G2<br>16 (27.12 %) G3	0 (0.00 %) G1<br>15 (71.33 %) G2<br>6 (28.57 %) G3	0.0729
Perineural invasion	53 (91.38 %)	19 (90.48 %)	0.9001
Microvascular invasion	42 (71.19 %)	14 (66.67 %)	0.6980
AbPas (+)	26 (44.07 %)	14 (66.67 %)	0.0753
Surgical margin status	35 (59.32 %) R0<br>12 (20.34 %) R1 direct<br>11 (18.64 %) R1<br>1 (1.69 %) R2	9 (42.86 %) R0<br>5 (23.81 %) R1 direct<br>5 (23.81 %) R1<br>2 (9.52 %) R2	0.3094
Weight [kg]	73.12 ± 11.31	64.74 ± 12.82	0.0098
Weight loss [kg]	4.93 ± 7.15	13.64 ± 7.69	0.0006
Weight loss<br>1. Stable	44 (75.58 %)	5 (23.81 %)	< 0.0001

2. Weight loss ≤ 10 %	8 (13.56 %)	4 (19.05 %)	
3. Weight loss > 10 %	7 (11.86 %)	12 (57.14 %)	
BMI groups<br>1. < 18.5<br>2. ≥ 18.5	 1 (1.69 %)<br>58 (98.31 %)	 2 (9.52 %)<br>19 (90.48 %)	0.1048
BMI groups<br>1. < 30<br>2. ≥ 30	 54 (91.53 %)<br>5 (8.47 %)	 21 (100.00 %)<br>0 (0.00 %)	0.1682
BMI groups according to WHO classification<br>< 18.5<br>18.5-24.9<br>25-29.9<br>≥ 30	 3 (3.75 %)<br>33 (41.25 %)<br>39 (48.75 %)<br>5 (6.25 %)	1 (1.69 %)<br>11 (52.38 %)<br>8 (38.10 %)<br>0 (0.00 %)	0.1201
BMI [kg/m²]	25.53 ± 3.46	23.66 ± 3.37	0.0358
Arterial hypertension	28 (47.46 %) No<br>31 (52.54 %) Yes	12 (57.14 %) No<br>9 (42.86 %) Yes	0.3060
Ischemic heart disease	54 (91.53 %) No<br>5 (8.47 %) Yes	21 (100 %) No<br>0 (0.00 %) Yes	0.1683
Diabetes mellitus (type 2)	41 (69.49 %) No<br>18 (30.51 %) Yes	14 (66.67 %) No<br>7 (33.33 %) Yes	0.5067
Smoking	37 (62.71 %) No<br>22 (37.29 %) Yes	11 (52.38 %) No<br>10 (47.62 %) Yes	0.2825
Alcohol consumption	53 (89.83 %) No<br>6 (10.17 %) Yes	19 (90.48 %) No<br>2 (9.52 %) Yes	0.6500
Duration of clinical manifestation [months]	4.55 ± 3.91	5.78 ± 3.86	0.2995
Duration of weight loss [months]	3.50 ± 3.12	4.50 ± 3.03	0.3014
Common clinical symptoms			
Jaundice	28 (47.00 %)	14 (67.00 %)	0.1035
Diarrhea	9 (15 %)	4 (19.00 %)	0.4609
Abdominal pain	26 (44.00 %)	11 (52 %)	0.3434
Neoadjuvant chemotherapy	5 (8.00 %)	7 (33.00 %)	0.0061
Count of chemotherapy cycles	6.00 ± 1.09	8.20 ± 4.32	0.2561
Preoperative biliary stenting	28 (47.46 %) No<br>31 (52.54 %) Yes	5 (23.81 %) No<br>16 (76.19 %) Yes	0.0492
Duration of hospitalization [days]	13.89 ± 5.83	17.24 ± 14.35	0.6658
Postoperative morbidity rate	11 (20.00 %)	7 (35.00 %)	0.1495
Postoperative mortality rate	0 (0.00 %)	1 (5.00 %)	0.0980
Reoperation rate	6 (10.00 %)	3 (14.00 %)	0.4361
Readmission rate	4 (7.00 %)	1 (5.00 %)	0.6260
Duration of operation	430.15 ± 125.47	504.95 ± 120.20	0.0201
Blood loss [ml]	520.21 ± 301.92	553.33 ± 451.77	0.7799
ASA classification<br>1.<br>2.<br>3.	 1 (2.0 %)<br>25 (42.00 %)<br>33 (65 %)	 0 (0.00 %)<br>12 (57.00 %)<br>9 (43.00 %)	0.4514
Smoking	37 (62.71 %) No<br>22 (37.29 %) Yes	11 (52.38 %) No<br>10 (47.62 %) Yes	0.4066

Postoperative parenteral nutrition	52 (88.14 %) No<br>7 (11.86 %) Yes	14 (66.67 %) No<br>7 (33.33 %) Yes	0.0262
Duration of parenteral nutrition [days]	4.71 ± 5.59	10.62 ± 6.41	0.0349
Total protein [g/dl]	6.18 ± 1.07	5.86.1.33	0.2248
Albumin [g/dl]	3.56 ± 6.80	3.35 ± 8.77	0.3478
White blood cell count [/mm³]	7.23 ± 2.05	7.88 ± 2.46	0.2401
Neutrophil count [/mm³]	4.47 ± 1.78	4.41 ± 0.93	0.9025
Monocyte count [/mm³]	0.62 ± 0.22	0.61 ± 0.33	0.2690
Total lymphocyte count [/mm³]	1.88 ± 0.62	1.58 ± 0.90	0.0292
Platelet count[/mm³]	254.18 ± 104.82	257.86 ± 99.60	0.8243
Hemoglobin [g/dl]	13.10 ± 1.53	13.04 ± 1.55	0.8693
CRP [mg/l]	8.15 ± 11.92	4.93 ± 5.79	0.4262
ALT [U/l]	33.95 ± 21.71	37.35 ± 19.45	0.6125
AST [U/l]	41.00 ± 39.44	38.59 ± 25.20	0.9790
GGT [U/l]	49.63 ± 46.37	68.84 ± 49.70	0.4019
ALP [U/l]	113.85 ± 39.54	132.10 ± 58.88	0.3210
Bilirubin [g/dl]	1.14 ± 0.70	1.10 ± 1.07	0.5157
Ca 19.9 [U/ml]	227.65 ± 228.95	652.82 ± 831.53	0.5261
CEA [ng/ml]	3.87 ± 2.86	14.79 ± 32.64	0.0275
PNI	46.03 ± 8.47	41.91 ± 7.74	0.1111
NLR	2.45 ± 1.24	3.11 ± 2.13	0.1815
MLR	0.35 ± 0.20	0.38 ± 0.15	0.2796
PLR	154.76 ± 97.43	163.53 ± 98.87	0.7505

NRS 2002, Nutritional Risk Score; BMI, body mass index; ASA, American Society of Anesthesiologists; ICU, Intensive Care Unit; ALT; alanine aminotransferase; AST, aspartate aminotransferase; GGT; gamma-glutamyl transpeptidase; ALP, alkaline phosphatase; CRP, C-reactive protein; APTT; activated partial thromboplastin time; INR; international normalized ratio; CEA, carcinoembryonic antigen; CA 19.9, carbohydrate antigen; PNI, prognostic nutritional index; NLR, Neutrophil/lymphocyte ratio; MLR, Monocyte/lymphocyte ratio; PLR, Platelet/lymphocyte ratio. Significant results ($p < 0.05$) are highlighted in red print.

Table S8. Comparison of selected clinicopathological and nutritional parameters depending on PNI.

Feature	PNI < 45 (*n* = 43)	PNI > 45 (*n* = 37)	*p* value
Age [years]	67.25 ± 8.92	64.48 ± 9.63	0.3215
NRS 2002	2.41 ± 1.41	2.00 ± 1.00	0.4128
ASA	2.62 ± 0.49	2.43 ± 0.51	0.2649
Weight [kg]	68.79 ± 13.74	73.00 ± 11.21	0.2709
BMI [kg/m²]	24.83 ± 3.71	25.07 ± 3.01	0.8110
Weight loss [kg]	7.92 ± 8.87	10.36 ± 9.75	0.5052
Duration of hospitalization	19.29 ± 13.34	14.05 ± 7.22	0.0741
Total protein [g/dl]	5.28 ± 0.98	6.65 ± 0.47	<0.0001
Albumin [g/dl]	2.90 ± 0.60	4.01 ± 0.48	<0.0001
Total lymphocyte count [/mm³]	1.78 ± 0.78	2.04 ± 0.82	0.2914
Neutrophil count [/mm³]	4.72 ± 1.70	4.51 ± 1.97	0.7114
Monocyte count [/mm³]	0.67 ± 0.23	0.64 ± 0.28	0.5541
White blood cell count [/mm³]	7.74 ± 1.82	7.48 ± 2.33	0.5618
Platelet count [/mm³]	279.87 ± 124.84	225.86 ± 82.87	0.0995
Hemoglobin [g/dl]	12.70 ± 1.58	13.33 ± 1.51	0.1778
CRP	10.19 ± 14.57	5.96 ± 7.57	0.9249
CA 19.9	621.35 ± 599.95	181.54 ± 163.36	0.0741

CEA	6.63 ± 9.85	4.07 ± 2.95	0.7983
NLR	2.96 ± 1.73	2.53 ± 1.56	0.4220
MLR	0.43 ± 0.23	0.33 ± 0.12	0.0670
PLR	187.13 ± 117.39	131.98 ± 81.56	0.0637

PNI, prognostic nutritional index; NRS 2002, Nutritional Risk Score; BMI, body mass index; ASA, American Society of Anesthesiologists; ALT; CEA, carcinoembryonic antigen; CA 19.9, carbohydrate antigen; PNI, prognostic nutritional index; NLR, Neutrophil/lymphocyte ratio; MLR, Monocyte/lymphocyte ratio; PLR, Platelet/lymphocyte ratio. Significant results ($p < 0.05$) are highlighted in red print.

Table S9. Comparison of selected clinicopathological and nutritional parameters depending on presence of postoperative complications.

Feature	No complications	Complications	*p* value
Age	64.91 ± 8.80	66.61 ± 7.49	0.4627
Gender	29 (50.88 %) Male<br>28 (49.12 %) Female	8 (44.40 %) Male<br>10 (55.56 %) Female	0.6341
Weight	70.93 ± 12.68	70.92 ± 12.28	0.9970
Weight loss	7.32 ± 8.82	8.37 ± 6.54	0.4039
BMI	24.78 ± 3.44	25.72 ± 3.64	0.3211
BMI groups<br>< 18.5<br>≥ 18.5	 2 (3.51 %)<br>55 (96.49 %)	 1 (5.56 %)<br>17 (94.44 %)	0.6993
BMI groups<br>< 30<br>≥ 30	 55 (96.49 %)<br>2 (3.51 %)	 15 (83.33 %)<br>3 (16.67 %)	0.0511
BMI groups according to WHO classification<br>< 18.5<br>18.5-24.9<br>25-29.9<br>≥ 30	2 (3.51 %)<br>26 (45.61 %)<br>27 (47.37 %)<br>2 (3.51 %)	1 (5.56 %)<br>5 (27.78 %)<br>10 (55.56 %)<br>2 (11.11 %)	0.4130
NRS 2002	2.03 ± 0.94	2.50 ± 1.38	0.3032
NRS 2002 groups<br>1. < 3<br>2. ≥ 3	 44 (77.14 %)<br>13 (22.81 %)	 11 (61.11 %)<br>7 (38.89 %)	0.1786
Tumor location	40 (70.18 %) Proximal<br>17 (29.82 %) Distal	14 (77.78 %) Proximal<br>4 (22.22 %) Distal	0.5311
Duration of hospitalization [days]	12.17 ± 3.56	22.94 ± 15.05	0.0001
Duration of hospitalization in ICU [days]	1.33 ± 1.00	10.25 ± 8.09	0.0087
Duration of postoperative hospitalization (patients number)<br>< 10 days<br>> 10 days	38 (61.29 %)<br>24 (38.71 %)	4 (22.22 %)<br>14 (77.78 %)	0.0036
Postoperative parenteral nutrition	6 (10.53 %)	7 (38.89 %)	0.0056
Duration of parenteral nutrition [days]	2.64 ± 4.08	12.11 ± 5.01	0.0012
Neoadjuvant chemotherapy<br>No<br>Yes	 54 (87.10 %)<br>8 (12.90%)	 14 (77.78 %)<br>4 (22.22 %)	0.3297
Total protein [g/dl]	6.24 ± 0.94	5.75 ± 0.97	0.0181

Albumin [g/dl]	3.64 ± 0.68	3.26 ± 0.80	0.0673
Hemoglobin [g/dl]	13.12 ± 1.48	12.72 ± 1.57	0.3231
CRP [mg/l]	6.81 ± 10.19	8.33 ± 12.22	0.0464
White blood cell count [/mm³]	7.24 ± 2.16	7.70 ± 2.27	0.4950
Total lymphocyte count [/mm³]	1.96 ± 0.68	1.86 ± 0.99	0.6807
Neutrophil count [/mm³]	4.47 ± 1.72	4.45 ± 1.50	0.8770
Monocyte count [/mm³]	0.59 ± 0.21	0.69 ± 0.35	0.5902
Platelet count [/mm³]	252.15 ± 92.54	259.58 ± 117.76	0.7546
PNI	45.00 ± 8.03	42.12 ± 8.55	0.0552
NLR	2.58 ± 1.50	2.75 ± 1.63	0.8651
MLR	0.32 ± 0.11	0.46 ± 0.29	0.0725
PLR	143.00 ± 75.91	193.43 ± 138.29	0.3955

NRS 2002, Nutritional Risk Score; BMI, body mass index; ASA, American Society of Anesthesiologists; CRP, C-reactive protein; PNI, prognostic nutritional index; NLR, Neutrophil/lymphocyte ratio; MLR, Monocyte/lymphocyte ratio; PLR, Platelet/lymphocyte ratio.

Significant results ($p < 0.05$) are highlighted in red print.

Table S10. Comparison of the incidence of postoperative complications according to duration of postoperative hospitalization.

**Duration of postoperative hospitalization**	**< 10 days (*n* = 42)**	**> 10 days (*n* = 38)**	***p* value**
Complications			
No	38 (90.48 %)	24 (63.16 %)	0.0035
Yes	4 (9.52 %)	14 (36.84 %)	

Significant results ($p < 0.05$) are highlighted in red print.

Table S11. Comparison of selected clinicopathological, immune and nutritional parameters depending on the use of neoadjuvant chemotherapy.

**Feature**	**No neoadjuvant chemotherapy (*n* = 68)**	**Neoadjuvant chemotherapy (*n* = 12)**	***p* value**
Age	65.28 ± 8.60	66.33 ± 7.25	0.6904
Gender	34 (50.00 %) Male<br>34 (50.00 %) Female	6 (50.00 %) Male<br>6 (50.00 %) Female	1.0000
Weight	71.02 ± 12.88	70.33 ± 13.69	0.8660
Weight loss	7.03 ± 8.22	11.57 ± 8.44	0.1901
BMI	25.16 ± 3.53	24.37 ± 3.44	0.4762
BMI groups			
< 18.5	3 (4.41 %)	12 (100.00 %)	0.0000001
≥ 18.5	65 (95.59 %)	0 (0.00 %)	
BMI groups			
< 30	63 (92.65 %)	12 (100.00 %)	0.3320
≥ 30	5 (7.35 %)	0 (0.00 %)	
NRS 2002	2.03 ± 1.10 (1-5)	2.75 ± 0.96 (1-4)	0.0375
NRS 2002 groups			
1. < 3	54 (79.41 %)	5 (41.67 %)	0.0061
2. ≥ 3	14 (20.59 %)	7 (58.33 %)	
Tumor location	49 (72.06 %) Proximal<br>19 (27.94 %) Distal	9 (75.00%) Proximal<br>3 (25.00 %) Distal	0.8334
Tumor depth (T)	9 (13.24 %) T1<br>51 (75.00 %) T2	4 (33.33 %) T1<br>7 (58.33 %) T2	0.2195

	8 (11.76 %) T3	1 (8.33 %) T3	
	28 (41.18 %) T1-2<br>40 (58.82 %) T3	5 (41.67 %) T1-2<br>7 (58.33 %) T3	0.9746
Lymph node invasion (N)	9 (13.24 %) N0<br>18 (26.47 %) N1<br>41 (60.29 %) N2	0 (0.00 %) N0<br>4 (33.33 %) N1<br>8 (66.67 %) N2	0.4004
	9 (13.24 %) N0<br>59 (86.76 %) N+	0 (0.00 %) N0<br>12 (100.00 %) N+	0.1810
Distal metastasis (M)	62 (91.18 %)<br>6 (8.82 %)	10 (83.33 %)<br>2 (16.67 %)	0.4037
Histological type<br>Adenocarcinoma<br>Adenosquamous carcinoma	 63 (92.65 %)<br>5 (7.35 %)	 12 (100.00 %)<br>0 (0.00 %)	0.3320
Histological grading	7 (10.29 %) G1<br>42 (61.76 %) G2<br>19 (27.94 %) G3	0 (8.33 %) G1<br>15 (66.67 %) G2<br>6 (25.00 %) G3	0.9462
Duration of postoperative hospitalization [days]<br>< 10<br>> 10	 34 (50.00 %)<br>34 (50.00 %)	 8 (66.67 %)<br>4 (33.33 %)	0.2865
Duration of hospitalization in ICU [days]	4 ± 6.23 (0-22)	2 ± 0.00 (2-2)	0.7624
Postoperative parenteral nutrition	12 (17.65 %)	2 (16.67 %)	0.9343
Duration of parenteral nutrition [days]	6.45 ± 5.59 (0-15)	11 ± 15.56 (0-22)	0.3537
Postoperative complication rate	14 (20.59 %)	4 (33.33 %)	0.3297
Postoperative complications according to Clavien Dindo classification<br>0<br>1<br>2<br>3<br>4<br>5	 53 (77.94 %)<br>5 (7.35 %)<br>1 (1.47 %)<br>8 (11.76 %)<br>0 (0.00 %)<br>1 (1.47 %)	 9 (75.00 %)<br>2 (16.67 %)<br>1 (8.33 %)<br>0 (0.00 %)<br>0 (0.00 %)<br>0 (0.00 %)	0.3387
Reoperation rate	8 (11.76 %)	1 (8.33 %)	0.7287
Mortality rate	1 (1.47 %)	0 (0.00 %)	0.6725
Total protein [g/dl]	6.04 ± 0.97	6.44 ± 0.89	0.1933
Albumin [g/dl]	3.48 ± 0.74	3.64 ± 0.71	0.4875
Hemoglobin [g/dl]	13.15 ± 1.54	12.71 ± 1.46	0.3565
CRP [mg/l]	7.71 ± 11.37	4.58 ± 3.48	0.4195
White blood cell count [/mm^3]	7.25 ± 1.99	8.23 ± 2.95	0.1490
Total lymphocyte count [/mm^3]	1.91 ± 0.77	2.15 ± 0.81	0.4440
Neutrophil count [/mm^3]	4.42 ± 1.60	4.70 ± 1.77	0.6668
Monocyte count [/mm^3]	0.60 ± 0.22	0.76 ± 0.38	0.1128
Platelet count [/mm^3]	262.71 ± 107.39	213.00 ± 59.15	0.1238
PNI	44.78 ± 8.43	46.87 ± 8.76	0.5426
NLR	2.56 ± 1.36	2.89 ± 2.30	0.5970
MLR	0.36 ± 0.19	0.38 ± 0.16	0.7976

PLR	160.28 ± 96.13	131.54 ± 96.94	0.4619

NRS 2002, Nutritional Risk Score; BMI, body mass index; ASA, American Society of Anesthesiologists; CRP, C-reactive protein; PNI, prognostic nutritional index; NLR, Neutrophil/lymphocyte ratio; MLR, Monocyte/lymphocyte ratio; PLR, Platelet/lymphocyte ratio.
Significant results ($p < 0.05$) are highlighted in red print.
